# Duffy Antigen Receptor for Chemokine (DARC) Polymorphisms and Its Involvement in Acquisition of Inhibitory Anti-Duffy Binding Protein II (DBPII) Immunity

**DOI:** 10.1371/journal.pone.0093782

**Published:** 2014-04-07

**Authors:** Flávia A. Souza-Silva, Letícia M. Torres, Jessica R. Santos-Alves, Michaelis Loren Tang, Bruno A. M. Sanchez, Tais N. Sousa, Cor J. F. Fontes, Paulo A. Nogueira, Roberto S. Rocha, Cristiana F. A. Brito, John H. Adams, Flora S. Kano, Luzia H. Carvalho

**Affiliations:** 1 Laboratório de Malária, Centro de Pesquisas René Rachou, Fundação Oswaldo Cruz (FIOCRUZ), Belo Horizonte, MG, Brasil; 2 Universidade Federal de Mato Grosso, campus Sinop, Sinop, MT, Brasil; 3 Julio Müller University Hospital, Universidade Federal de Mato Grosso, Cuiabá, MT, Brasil; 4 Centro de Pesquisas Leônidas & Maria Deane, FIOCRUZ Amazônia, Manaus, AM, Brasil; 5 Department of Global Health, College of Public Health, University of South Florida, Tampa, Florida, United States of America; London School of Hygiene and Tropical Medicine, United Kingdom

## Abstract

The *Plasmodium vivax* Duffy binding protein (PvDBP) and its erythrocytic receptor, the Duffy antigen receptor for chemokines (DARC), are involved in the major *P. vivax* erythrocyte invasion pathway. An open cohort study to analyze *DARC* genotypes and their relationship to PvDBP immune responses was carried out in 620 volunteers in an agricultural settlement of the Brazilian Amazon. Three cross-sectional surveys were conducted at 6-month intervals, comprising 395, 410, and 407 subjects, respectively. The incidence rates of *P. vivax* infection was 2.32 malaria episodes per 100 person-months under survey (95% confidence interval [CI] of 1.92-2.80/100 person-month) and, of *P. falciparum*, 0.04 per 100 person-months (95% CI of 0.007–0.14/100 person-month). The distribution of DARC genotypes was consistent with the heterogeneous ethnic origins of the Amazon population, with a predominance of non-silent *DARC* alleles: FY*A > FY*B. The 12-month follow-up study demonstrated no association between *DARC* genotypes and total IgG antibodies as measured by ELISA targeting PvDBP (region II, DBPII or regions II–IV, DBPII-IV). The naturally acquired DBPII specific binding inhibitory antibodies (BIAbs) tended to be more frequent in heterozygous individuals carrying a *DARC*-silent allele (FY*B^ES^). These results provide evidence that DARC polymorphisms may influence the naturally acquired inhibitory anti-Duffy binding protein II immunity.

## Introduction

*Plasmodium vivax* is the most widespread *Plasmodium* species and is a potential cause of morbidity and mortality among the 2.48 billion people living at risk of infection [1]. Recent evidence of multidrug-resistant *P. vivax* associated with severe and fatal disease elevates it to one of global health concern [2], [3]. *Plasmodium vivax* infects human erythrocytes (RBCs) through a pathway that requires interaction between an apical parasite protein, the Duffy binding protein (PvDBP), and its receptor on reticulocytes, the Duffy antigen receptor for chemokines (DARC) [4]–[6]. The goal in developing PvDBP as a vaccine against blood stages of *P. vivax* is to elicit an antibody response that inhibits parasite adhesion to DARC-positive human reticulocytes, and thereby prevents merozoite invasion. The importance of the interaction between PvDBP (region II, DBPII) and DARC to *P. vivax* infection has stimulated a significant number of studies of PvDBP antibody responses. Available data demonstrate that naturally occurring antibodies to PvDBP are prevalent in individuals living in *P. vivax* endemic areas [7]–[9], and these antibodies can block the DBPII/DARC interaction [10]–[12]. While inhibitory DBPII antibodies confer a degree of protection against blood stage infection [12], these antibodies are biased towards a specific allele [13]. Although anti-PvDBP immune responses have been well characterized, little is known about the association between this immune response and DARC host genotype [14], [15].

Although most individuals lacking DARC on their RBCs are naturally resistant to *P. vivax* [4], some infections occur in DARC-negative persons living in vivax malaria endemic areas [16]–[18]. Beyond being receptors for *P. vivax* and various chemokines [19], DARC proteins have clinical and biological significance and have been reported to be associated with transfusion incompatibility and hemolytic disease of the newborn [20]–[22]. It is also implicated in several inflammatory diseases, and cancer, and might play a role in HIV infection and AIDS [23]–[26]. Recently, a previously unreported function of this receptor has been described in *P. falciparum* infection, in which DARC proteins seem to be essential for platelet-mediated killing of *P. falciparum* parasites [27].

The two common *DARC* alleles in Caucasians, FY*A and FY*B, differ by a single base substitution (125 G>A) resulting in the replacement at residue 42 in the extracellular domain of a glycine (Fya antigen) for an aspartic acid (Fyb antigen) [28], [29]. Another mutation in the DARC gene promoter region abolishes receptor expression on erythroid cells by disruption of a binding site for the GATA1 erythroid transcription factor, resulting in the absence of DARC antigens on RBCs (-33T>C; Fy*^ES^*, erythrocyte-silent) [30]. Although most DARC negative individuals carry the GATA mutation in the FY*B allele (silent FY*B allele), the presence of a *cis*-regulatory mutation within FY*A has been described [31]. The overall expression level of erythroid-specific DARC is co-dominant; therefore, DARC-null promoter heterozygosity reduces the DARC expression level by approximately 50 percent [31]–[33]. Similarly, the susceptibility to *P. vivax* in DARC-positive individuals varies among specific *DARC* genotypes [31], [34]–[36].

In the current study, we present data of the first population-based study of the relationship between DARC genotypes and PvDBP inhibitory antibodies. The methodology included a community-based open cohort study in an agricultural settlement of the Amazon area of Brazil in which 620 individuals were genotyped for DARC, and their PvDBP immune responses were evaluated by conventional serology (recombinant proteins) and binding inhibitory antibodies (BIAb) targeting the DBPII ligand.

## Material and Methods

### Study area and population

The study was carried-out in the agricultural settlement of Rio Pardo (1°46′S–1°54′S, 60°22′W–60°10′W), Presidente Figueiredo municipality, northeast Amazonas State in the Brazilian Amazon area. Rio Pardo is located approximately 160 km from Manaus, the capital of Amazonas, along the main access to a paved road (BR-174) that connects Amazonas to Roraima State. The settlement was officially created in 1996 by the National Institute of Colonization and Agrarian Reform (INCRA) as part of a large scale colonization project focused on agriculture and wide-ranging human settlement in the Amazon area [37]. The mean annual temperature is 31°C with humid climate and average annual rainfall of the 2,000 mm per year. The rainy season extends from November-May and dry season from June–October. The settlement is composed of areas called ‘ramais’, which include households on both sides of unpaved roads, and a riverine population called Igarapé. A census in September–October 2008 identified 701 inhabitants, with 360 (51.4%) living in ramais areas and 341 (48.6%) in and around Igarapé. Inhabitants of the area live on subsistence farming and fishing along the small streams of the Rio Pardo River. The study site and malaria transmission patterns have been described in detail elsewhere [38]. Although *P. vivax* and *P. falciparum* are transmitted year round, *P. vivax* is responsible for about 90% of malaria cases [38]. Housing quality is poor, rendering indoor residual spraying ineffective. The availability of curative services is limited, and a government outpost provides free malaria diagnosis and treatment.

### Study design and cross-sectional surveys

The ethical and methodological aspects of this study were approved by the Ethical Committee of Research on Human Beings from the Centro de Pesquisas René Rachou (Report No. 007/2006 and No. 07/2009), according to the Resolution of the Brazilian Council on Health-CNS 196 / 96 after consultation with the community. In November of 2008, of 701 residents of the settlement invited to participate in the study, 541 (77.2%) accepted by giving written informed consent, which was also obtained from the next of kin, caregivers, or guardians on the behalf of participating minors. In addition to the consent form, separate assent forms were obtained from minors, in language appropriate for children ages 7–13 and adolescents from 14 to 17 years of age.

A population-based open cohort study was initiated in November of 2008, with the following procedures [38]: (i) administration of a structured questionnaire to all volunteers to obtain demographical, epidemiological, and clinical data; (ii) physical examination, including body temperature and spleen/liver size, recorded according to standard clinical protocols; (iii) venous blood collection in individuals aged five years or older (EDTA 10 ml), or blood spotted on filter paper (finger-prick) in those aged <5 years; and (iv) search for malaria parasites by light microscopy on Giemsa thick blood smears. Geographical location of each dwelling was recorded using a hand-held 12-channel global positioning system (GPS) (Garmin 12XL, Olathe, KS, USA) with a positional accuracy within 15 m. At initial enrollment, of 541 volunteers, genomic DNA was amplified to *DARC* polymorphisms and plasma samples screened to *P. vivax* antibodies in 395 (73%).

Six and twelve months following the initial survey, identical cross-sectional surveys were carried out. In total, 395 subjects were enrolled at baseline, 410 at the 2nd survey (June 2009), and 407 in the 3^rd^ survey (October-November, 2009). A total of 620 volunteers contributed DNA and plasma samples, some of which participated in more than one cross-sectional survey. One hundred eighty-two (29.4%) and 205 (33%) subjects provided samples in two and three cross-sectional surveys, respectively.

### Laboratory diagnosis of malaria

Malaria infection was diagnosed by microscopy of Giemsa-stained thick smears and real-time PCR amplification of a species-specific segment of the multicopy 18S rRNA gene of human malaria parasites. The Giemsa-stained smears were evaluated by experienced microscopists, according to the malaria diagnosis guidelines of the Brazilian Ministry of Health. For real-time PCR, genomic DNA was extracted from either EDTA whole-blood samples (adults and children≥5 years old) or dried blood spots on filter paper (children<5 years) using the Puregene blood core kit B (Qiagen, Minneapolis, MN, USA) or the QIAmp DNA mini kit (Qiagen), respectively, according to manufacturers' instructions. Real-time PCR was performed as previously described [39] using a consensus pair of primers (PL1473F18 [5′- TAACGAACGAGATCTTAA-3′] and PL1679R18 [5- GTTCCTCTAAGAAGC TTT-3′]).

### DARC genotyping

Extracted genomic DNA was used to detect the three common alleles at the *DARC* locus, FY*A, FY*B, and FY*B^ES^ (ES =  erythroid silent), using real-time PCR with allele-specific primers, as previously described with minor modification [34]. Essentially, the original FGATA and RYA primers were replaced to FGATANEW (5′ CCC GGG CCC GCC GCC CTCA TTA GTC CTT GGC TCT TGC 3′) and RYANEW (5′ AG CTG CTT CCA GGT TGG CGC 3′), respectively. Each 20 μl reaction mix contained 50–100 ng genomic DNA, 10 μL SYBR Green PCR master mix (Biosystems) and 0.1–1.0 pmoles/μL of each primer (Biosystems). The amplification and fluorescence were detected by ABI Prism 7000 Sequence Detection System (Applied Biosystems) using a cycle of 95°C for 10 min, followed by 35 cycles of 95°C for 15 s and 60°C for 1 min. After amplification, melting curves were observed from the dissociation curve resulting from continuous measurements of fluorescence (F) at 530 nm during which the temperature was gradually increased from 60 to 95°C. Melting peaks of each amplified fragment were visualized by plotting the negative derivative of the fluorescence over temperature versus temperature (–dF/dT° vs. T°). DNA samples from previously well-characterized Brazilian individuals carrying the most common DARC genotypes were included as positive controls. In addition, in c. 10% of samples, a 942-bp fragment of DARC, comprising polymorphic positions −33T>C and 125G>A, was amplified by PCR using the forward primer: 5′-TCAAAACAGGAAGACCCAAG-3′ and reverse: 5′-AGAGGTCTGAAAAGCATGAA-3′ (Macrogen DNA sequencing services; http://dna.macrogen.com/eng/). No discordant results were obtained.

### Recombinant protein and serological assay

The enzyme-linked immunosorbent assay (ELISA) for total IgG antibodies to PvDBP was carried out using two recombinant proteins, covering the region II (DBPII) or regions II–IV (DBPII-IV). The recombinant DBPII, which includes amino acids 243–573 (regions II), was expressed as a 6xHis fusion protein of 39 kDa, as previously described [40]. The recombinant DBPII-IV, amino acids 132–771 (regions II–IV), was expressed as a soluble glutathione S-transferase (GST) fusion protein of 140 kDa, as previously described [7], [8]. To assess IgG antibodies to PvDBP, ELISA was carried out as previously described [8] with serum samples at 1∶100 and recombinant proteins at a final concentration of 3 μg/mL (DBPII) or 1.25 μg/mL (DBPII-IV). For DBPII-IV protein, the final optical density (OD) at 492 nm was calculated by subtracting the OD obtained with GST (antigen control). The results were expressed as reactivity index: RI  =  OD values of test sample divided by the value of the cut-off. For each recombinant protein, cut-off points were set at three standard deviations above the mean OD of sera from 30 individuals who had never been exposed to malaria. Values of RI>1.0 were considered positive. For statistical purposes, individuals were categorized as high responders (RI>4) or low responders (RI≤4). This criterion was defined based on our previous findings that few individuals develop PvDBP antibody at RI>4 [38].

### COS cell transfection and erythrocyte-binding assays

COS7 (green monkey kidney epithelium, ATCC, Manassas, VA) cells were transfected with the plasmid pEGFP-DBPII, which coded for a common DBPII sequence circulating in the Amazon area [38]. This construct allows expression of DBPII as a fusion protein to the N-terminus of EGFP used as a transfection marker as previously described [10]. Transfections were realized with lipofectamine and PLUS-reagent (Invitrogen Life Technologies, Carlsbad, CA) according to manufacturer's protocols. Briefly, COS-7 cells in six-well culture plate (1.5×10^5^ cells/well) were transfected with plasmid (0.5 μg/well)-liposome complexes (5% Plus-reagent and 3% lipofectamine) in Dulbecco's Modified Eagle's Medium (DMEM, Sigma, St. Louis, MO) without serum. After 6 h exposure to DNA-liposome complexes (37°C, 5% CO_2_), transfection medium was replaced with DMEM with 10% fetal bovine serum (Gibco-BRL, Gaithersburg, MD). At 24 h post-transfection, culture medium was again replaced and efficiency of transfection was assessed by fluorescence. Forty-eight hours post-transfection, erythrocyte-binding assays were performed as previously described [11]. Briefly, plasma samples were added at 1∶40, and plates were incubated for 1 hr at 37°C in 5% CO_2_. Human O^+^ DARC^+^ erythrocytes in a 10% suspension were added to each well (200 μL/well), and plates were incubated for 2 h at room temperature. Since *DARC* genotypes might influence *in vitro* erythrocyte-binding assays [36], as was confirmed here (Figure S1), all *in vitro* binding assays were carried-out with erythrocytes expressing both DARC antigens (Fya and Fyb, FY*A/FY*B genotype), which display intermediate binding and predominate in Brazil. After incubation, unbound erythrocytes were removed by washing the wells three times with phosphate buffered saline (PBS). Binding was quantified by counting rosettes observed in 10–20 fields of view (200×). Positive rosettes were defined as adherent erythrocytes covering more than 50% of the COS cell surface. For each assay, pooled plasma samples from Rio Pardo residents characterized as non-responders by ELISA were used as a negative control (100% binding). For this purpose, only plasma that did not inhibit erythrocyte binding, as opposed to samples from unexposed Brazilian donors, was pooled for the negative control (usually, 10 plasma samples/pool). A positive control included a pool of plasma from individuals with long-term exposure to malaria in the Amazon area. The percent inhibition was calculated as 100 × (Rc - Rt)/Rc, where Rc is the average number of rosettes in the control wells and Rt is the average number of rosettes in the test wells.

### Statistical analysis

A database was created with Epidata software (http://www.epidata.dk). Linear correlations between two variables were determined by using the Pearson correlation coefficient. Comparisons of two independent proportions were realized by Z-test or chi-square test as appropriate. McNemar's test was used for analysis of correlated proportions. Differences in medians were assessed using Kruskal–Wallis with Dunn's post hoc test to identify differences between groups. The 95% Mid-P exact test was used to estimate incidence ratios (person-time of follow-up) and confidence intervals, considering variables such as duration of malaria-exposure, number of persons exposed, and previous malaria episodes. The level of significance of 5% was adopted. All analyses were performed on Stata software, v12 and OpenEpi (http://www.openepi.com/v37/Menu/OE_Menu.htm).

## Results

### Malarial infection, enrollment and follow-up, and PvDBP conventional serology

We investigated acute malaria infection in 620 subjects with a median of age 28 years and 1.4∶1 male-female (Table 1). Age of subjects basically corresponded to the time of malaria exposure in the Amazon area (r = 0.91; P<0.0001, Pearson's correlation test). Median time of residency in the Amazon area was 24 years. At the time of first blood collection, 19 (3%) subjects had positive blood-smears, with 17 (90%) of these infections caused by *P. vivax*. Real-time PCR confirmed all microscopically positive samples. In addition, PCR-based protocol identified 17 additional malaria infections, 15 *P. vivax* and two *P. falciparum*. The overall prevalence of malaria was 5.8%, with 32 of the 36 (89%) infections caused by *P. vivax*. No *P. malariae* or mixed *Plasmodium* infections were diagnosed by either microscopy or real-time PCR.

**Table 1:** Demographic, epidemiological and genetic data of 620 subjects, Rio Pardo Settlement, Amazonas, Brazil.

Characteristic	
**Median age, years (IQR)**	28 (14–47)
**Gender, male∶female**	1.4∶1
**Acute malaria infection, n (%)**	
Light Microscopy (LM)	19 (3.1)
PCR	17 (2.7)
Total	36 (5.8)
**Years of malaria exposure, median (IQR)**	24 (13–41)
**Previous malaria episodes, median (IQR)**	5 (1–11)
***DARC*** ** genotypes, n (%)**	
*FY* * *A/FY* * *B*	182^a^ (29.4)
*FY* * *A/FY* * *A*	137^b^ (22.1)
*FY* * *A/FY* * *B^ES^*	125^b^ (20.2)
*FY* * *B/FY* * *B*	87^c^ (14.0)
*FY* * *B/FY* * *B^ES^*	69^d^ (11.1)
*FY* * *B^ES^/FY* * *B^ES^*	20^e^ (3.2)
***DARC*** ** alleles, n (%)** *	
*FY* * *A*	581 (46.8)
*FY* * *B*	425 (34.3)
*FY* * *B^ES^*	234 (18.9)

IQR  =  interquartile range.

LM: 17 P. vivax, two P. falciparum; Real-Time PCR: 15 P. vivax, two P. falciparum.

a–e Significant differences (P<0.05, Z-test).

* Proportions differ significantly, FY*A> FY*B> FY*B^ES^, (P < 0.0001, Z-test).

**Figure 1:**
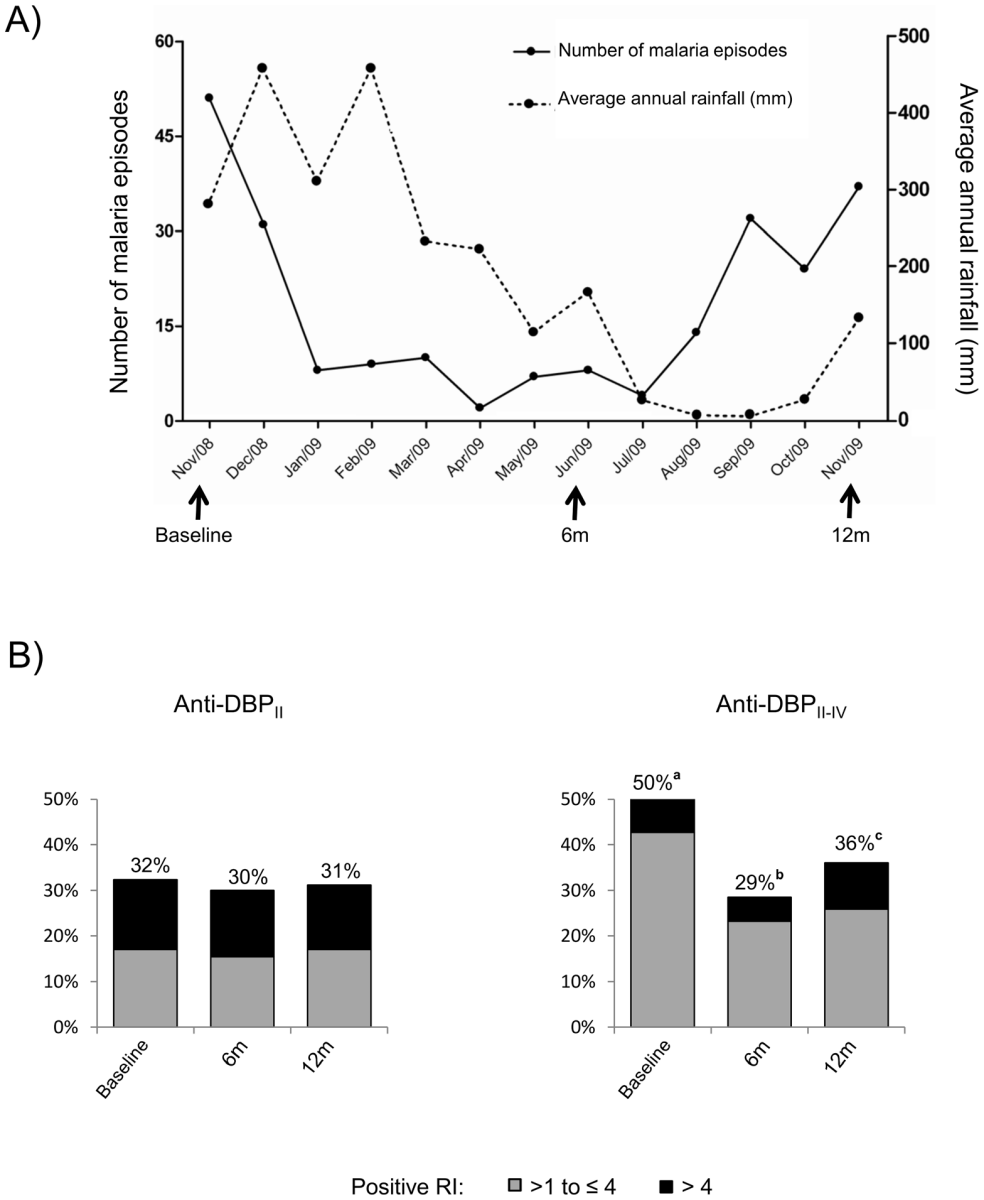
Temporal distribution of *P. vivax* malaria episodes and serological evaluation of ELISA-detected IgG antibodies targeting PvDBP, Amazonas, Brazil (Nov 2008-Nov 2009). (A) Episodes of malaria, as detected by conventional microscopy, varied according to rainfall and season; (B) DBPII-IV antibodies fluctuate with season while anti-DBPII remains stable. ELISA-detected antibody responses were evaluated November 2008, June 2009, and November 2009. Results are expressed as frequency (%) of responders, with Reactivity Index (RI)>1.0 considered positive; different superscripts (a–c) indicate significant differences (P<0.05 by Z-test.)

The 620 participants were followed up for an average of 7 months (10 days to 12 months), thus representing 4,646 person-months of follow-up. Based on parasitological-confirmed cases, the incidence rates of *P. vivax* malaria was 2.32 episodes per 100 person-months (95% confidence interval [CI] of 1.92–2.80/100 person-month) and, of *P. falciparum* 0.04 per 100 person-months (95% CI of 0.007–0.14/100 person-month). The temporal distribution of vivax malaria episodes according to rainfall and season is illustrated in Figure 1A.

Among the 620 recruited volunteers, 395 were enrolled at the baseline, 410 during the 2nd survey (6 months) and 407 during the 3rd survey (12 months). At the baseline, ELISA IgG antibodies to the main variant of PvDBP circulating in the area (Sal-1) were detected in 32% (region II, DBPII) to 50% (region II–IV, DBPII-IV) of the studied population (Figure 1B). The profile of DBPII antibody response was relatively stable over the course of the cross-sectional studies, and was not associated with the malaria transmission season (Figure 1B). On the other hand, DBPII-IV antibodies fluctuated according to malaria transmission (50% vs. 29% vs. 36% at baseline, 6, and 12 months, respectively; P<0.05 by Z-test). Consecutive serological surveys demonstrated that, while 50% of DBPII responders could be classified as high responders (RI>4), the majority of DBPII-IV responders were low responders (70 to 85%, RI≤4). In this area, presence of PvDBP antibodies seemed not to be influenced by acute malaria infection, as the frequency of responders was similar in infected and non-infected groups (P>0.05; Figure S2). However, the low numbers of acute infections identified in the study preclude conclusions about the association between infection and PvDBP antibody response. Acute infections were not explored in the further analyses.

### DARC polymorphisms and generation of *P. vivax* specific antibodies

Three *DARC* genotypes, FY*A/FY*B, FY*A/FY*A and FY*A/FY*B^ES^, were frequently observed (Table 1), with heterozygous FY*A/FY*B being the most prevalent (182 out of 620, 29.4%). The *DARC* negative genotype (FY*B^ES^/FY*B^ES^) was present at low frequency (3.2%, 20 of 620). Accordingly, we found a significant predominance of non-silent *DARC* alleles in the study area, with FY*A > FY*B > FY*B^ES^ (*p*<0.0001 by Z-test). Individuals carrying the different *DARC* genotypes were equally distributed among the demographical and epidemiological variables associated with the risk of malaria infection, such as age, sex, and dwelling location (data not shown).

### Anti-PvDBP IgG antibodies by conventional serology

**Figure 2:**
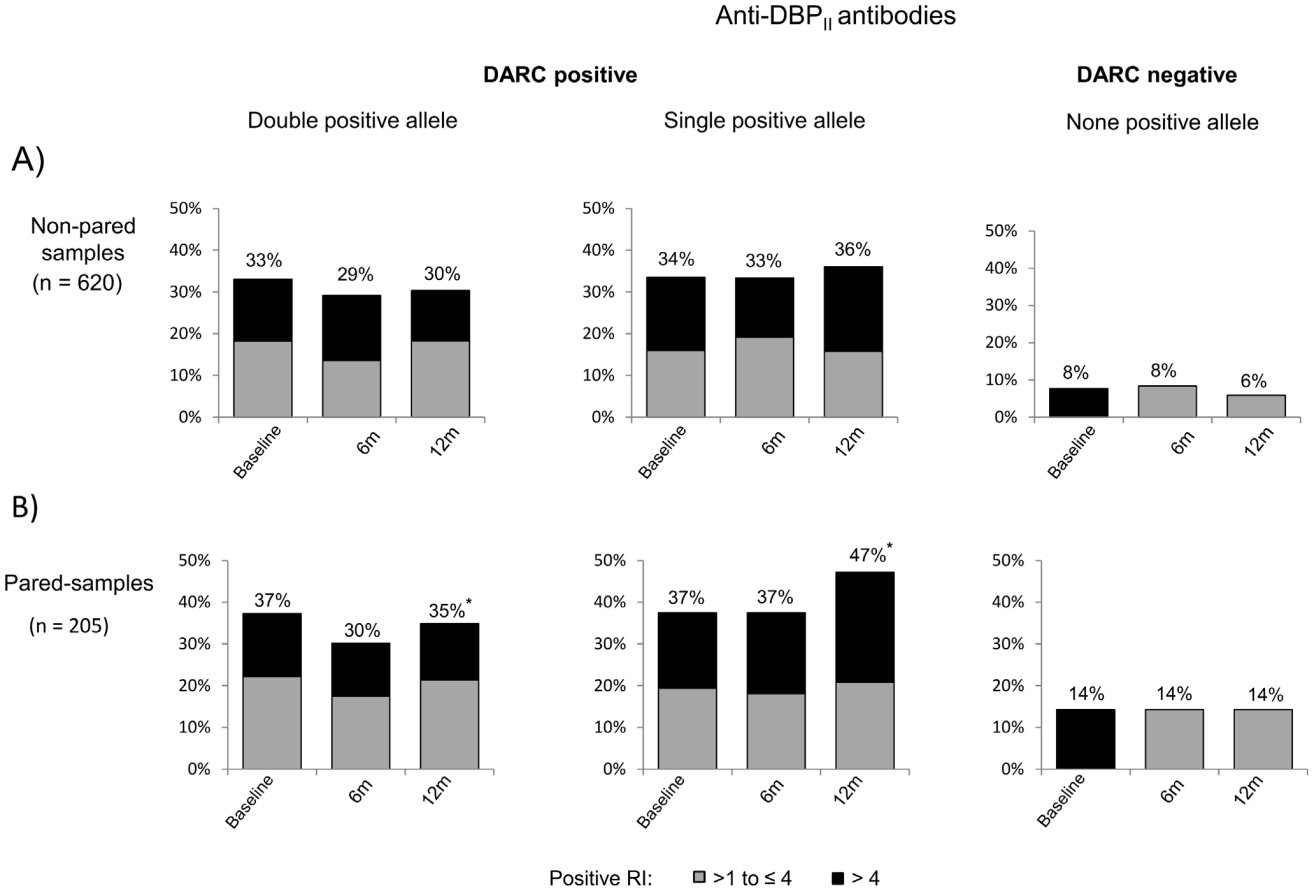
Frequencies of DBPII IgG ELISA-detected antibodies are similar in individuals carrying one or two *DARC* positive alleles. DBPII antibody responses were evaluated in plasma samples from individuals who participated in (A) at least one cross-sectional survey (non-paired samples, n = 620) or (B) in three consecutive surveys (paired-samples, n = 205). DARC positive individuals were categorized as carrying double positive alleles (FY*A/FY*A, FY*A/FY*B, FY*B/FY*B) or a single positive allele (FY*A/FY*B^ES^, FY*B/FY*B^ES^). DARC-negatives do not express DARC antigens on erythrocyte surface (FY*B^ES^/FY*B^ES^). ELISA results were expressed as frequency of responders (percentages on the top of the figures), with Reactivity Index (RI)>1.0 considered positive. For all frequency comparisons, significant differences were found only between DARC negative and DARC positive groups (P<0.05 by chi-square test).

To determine whether DARC polymorphisms influence the PvDBP antibody responses, we initially analyzed PvDBP antibodies as detected by ELISA. DBPII antibodies were analyzed at baseline and after 6 and 12 months, with individuals stratified according to *DARC* alleles, i.e., those carrying two (FY*A/FY*A, FY*A/FY*B, FY*B/FY*B), one (FY*A/FY*B^ES^, FY*B/FY*B^ES^), or no (FY*B^ES^/FY*B^ES^) *DARC*-positive allele (Figure 2). The frequency of responders among DARC negative individuals (FY*B^ES^/FY*B^ES^) was low, and their pattern of response remained the same at all survey times (Figure 2A). In DARC positive individuals, DBPII antibodies were detected in 29–36%, with no difference between individuals carrying one or two *DARC*-positive alleles (Figure 2A). A similar profile of response was detected for DBPII-IV antibodies (data not shown).

Since 205 of 620 individuals provided plasma samples at all three surveys (paired-samples), we analyzed this sub-group for differences in their anti-DBPII serological responses (Figure 2B). In general, the profiles of the DBPII antibody responses were similar between paired and non-paired samples (Figure 2). However, in the paired sub-group, it was possible to detect a slight trend toward a more intense response to DBPII among DARC positives carrying a single positive allele (high responder, RI>4.0) (Figure 2B). However, the difference was significant only at 12 months (Z-test = 3.2; P = 0.0014), with 35% and 47% responders detected among double and single positive carriers, respectively. This tendency was not associated with any particular genotype and was not detected by using another recombinant (DBPII-IV) (data not shown).

### DBPII binding inhibitory antibodies (BIAbs)

**Figure 3:**
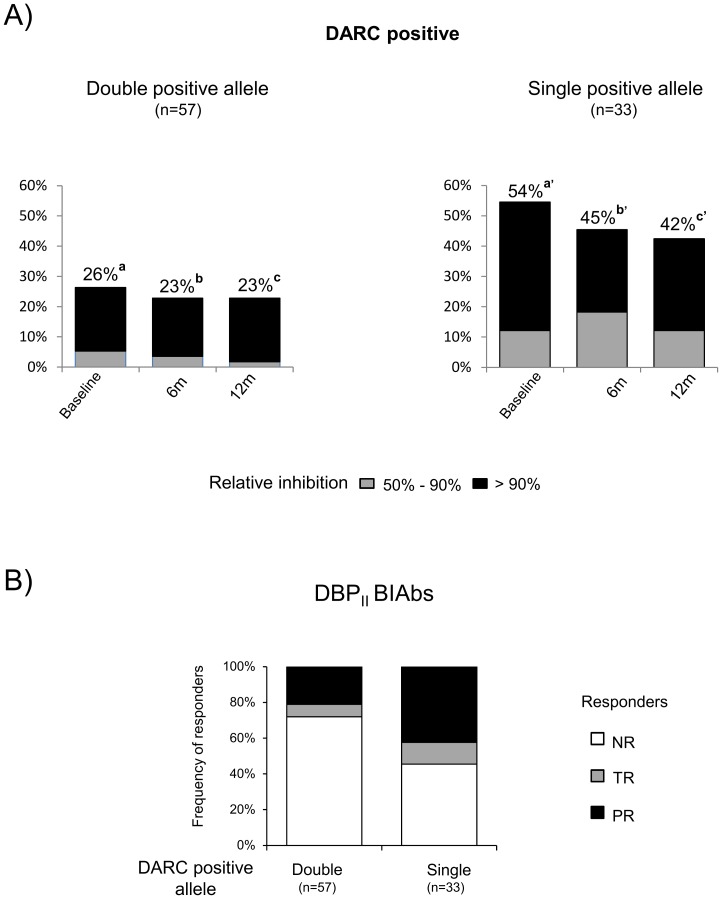
DARC polymorphisms affect the functional properties of DBPII antibodies. DBPII binding inhibitory antibodies (BIAbs) were evaluated in plasma from individuals stratified according to *DARC* alleles, as carrying double positive alleles (FY*A/FY*A, FY*A/FY*B, FY*B/FY*B) or a single positive allele (FY*A/FY*B^ES^, FY*B/FY*B^ES^). A) Frequencies of BIAbs (top of the columns) at three consecutive surveys showed DBPII BIAbs to be significantly more frequent in single positive allele carriers than in double positive carriers (significant differences between a *vs.* a′; b *vs.* b′ and c *vs.* c′, as determined by by Z-test, P<0.05.); B) After 12-months, the BIAbs response was more persistent in the group carrying a single *DARC*-positive allele (Z-test = 3.002; P = 0.0027). NR  =  non-responder, no inhibitory antibody response detected at any time-point of the study; TR  =  temporary responder, inhibitory antibodies in at least one cross-sectional survey; (PR  =  persistent responder; inhibitory antibodies detected in all surveys. BIAbs were evaluated by COS-7 cyto-adherence assay, with plasma samples at 1∶40 and positive results as DBPII –DARC binding inhibition ≥ 50%.

Further experiments investigated whether DARC polymorphisms altered the functional proprieties of DBPII antibodies. Due to the methodology constraints of performing functional assays, the three cross-sectional measures of DBPII BIAbs responses were performed on a subset of the study population comprising 270 samples corresponding to 90 DARC positive individuals (paired-samples) matched for age, sex, and malaria exposure (16 to 23 volunteers per *DARC*-positive genotype). Plasma samples from DARC negative individuals (n = 21) were used as a negative control, and no DBPII BIAbs could be detected in this group (data not shown). DBPII BIAbs were significantly more frequent in single *DARC*-positive allele carriers than in double positive carriers (Figure 3A). This pattern of acquired immune response was similar in all cross-sectional measures of DBPII BIAbs. Beyond the frequency and levels of inhibitory antibodies, the persistence of response was also higher in the group carrying a single *DARC*-positive allele (Z-test = 3.002; P = 0.0027) (Figure 3B), a result that was not detected by conventional serology (data not shown).

**Figure 4:**
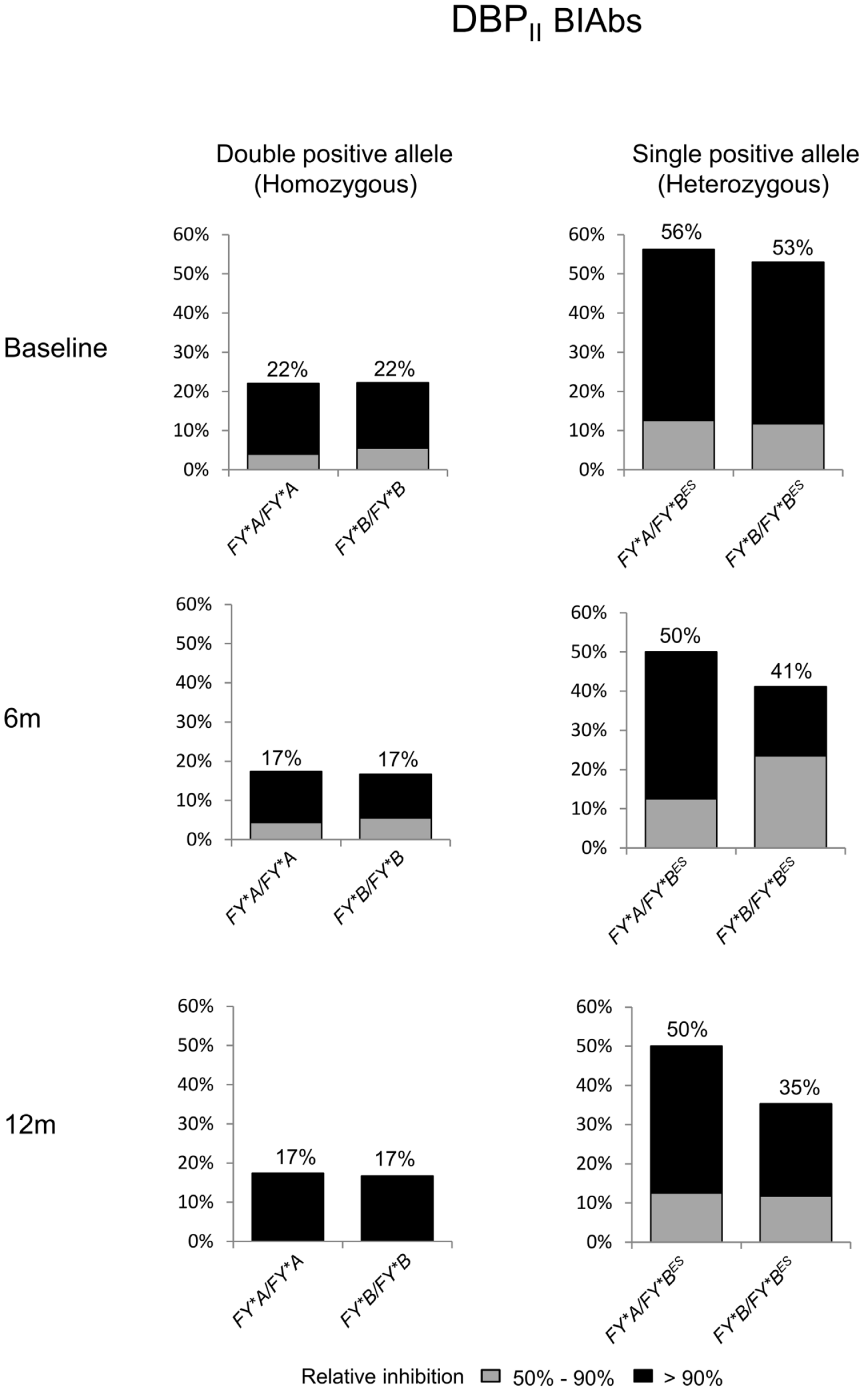
Homozygosis for either the FY*A or FY*B allele is associated with low frequency of DBPII BIAbs. Plasma samples were grouped according to *DARC* genotype, as described in legend of Figure 3. DBPII BIAbs were evaluated at three consecutive surveys by COS-7 cyto-adherence assay, with plasma at 1∶40 and DBPII –DARC binding inhibition ≥ 50% considered positive inhibition.

The tendency towards an increased DBPII inhibitory antibody response among single *DARC*-positive allele carriers led us to investigate whether a particular *DARC* genotype or allele could be associated with this response. Persons homozygous for either FY*A or FY*B alleles were associated with lower DBPII BIAbs; whereas the presence of these alleles in heterozygosis for the *GATA* mutation (FY*A/FY*B^ES^ or FY*B/FY*B^ES^) was associated with a much higher inhibitory response, and these profiles were consistent in all three surveys (Figure 4). Individuals heterozygous for *DARC*-positive alleles (FY*A/FY*B) developed an intermediate level of DBPII BIAbs (c. 38%). These results demonstrated that *DARC* genotype influences the frequency and stability of human DBPII inhibitory antibody response.

## Discussion

An important goal of PvDBP vaccine efforts is to inhibit parasite invasion of DARC positive reticulocytes. Since DARC polymorphisms are suspected to affect the ability of PvDBP antibodies to block parasite invasion [36], we carried-out the first follow-up population-based study of the relationship between DARC polymorphisms and DBP antibodies. In the study area, incidence rates for *P. vivax* and *P. falciparum* malaria were 2.32/100 and 0.04/100 person-months, respectively, allowing the classification of the area as hypo- to meso-endemic, consistent with the general profile of malaria transmission in the Amazon region [41]. At enrollment, 32% of the studied individuals showed ELISA-detected IgG antibodies to the ligand region II (DBPII), while a higher seroprevalence (50%) was found by using a recombinant protein covering regions II to IV (DBPII-IV). Although DBPII antibodies were less frequent than DBPII-IV antibodies, the magnitude of the DBPII immune response was significantly higher and included a significant number of high responders (RI>4), possibly because dominant B-cell epitopes lie on region II [42]–[44]. Accordingly, the analysis of consecutive serological surveys demonstrates that, while DBPII-IV antibodies fluctuate with season, DBPII antibodies were stable throughout the study period.

It is well known that DARC-negative individuals are highly resistant to *P. vivax* infection [4] and, in general, present only low levels of antibodies to *P. vivax* blood stages [14]. Therefore, we were particularly interested in investigating the influence of *DARC*-positive alleles on the acquisition of anti-PvDBP immunity. Among *DARC*-positive allele carriers, the frequencies of ELISA-detected IgG antibodies were similar in single (FY*A/FY*B^ES^ and FY*B/FY*B^ES^) and double positive (FY*A/FY*A, FY*B/FY*B and FY*A/FY*B) allele carriers. We observed a weak trend toward increased DBPII IgG antibodies among single positive allele carriers, although this association was not significant in two of the three cross-sectional surveys.

Few studies have investigated the influence of DARC-positive alleles on the presence of conventional PvDBP antibodies acquired post-infection [15], [36]. Using the recombinant DBPII expression construct used in the present study, but ELISA assays with plasma samples twice more concentrated, Maestre et al. [15] reported that Colombian subjects with one positive allele were more likely to show DBPII antibodies than were those with two positive alleles. Unfortunately, the low number of ELISA-positive individuals in the Colombian study (DBPII IgG sera, n = 17) precludes solid conclusions with respect to an association between PvDBP antibodies and the *DARC*-positive allele. In accordance with our findings, King et al. [36] reported that *DARC* genotypes were not associated with significant differences in DBPII ELISA-specific antibody responses. In conclusion, we found no clear association between *DARC*-positive alleles and conventional anti-PvDBP immune responses, as detected by ELISA using different recombinant proteins.

In contrast to conventional serology, the present study demonstrated that the frequency and magnitude of DBPII inhibitory antibody responses were significantly lower in adults carrying two *DARC*-positive alleles, especially in individuals homozygous for either the FY*A or the FY*B allele. Twelve months later, the frequency of persistent DBPII BIAb responders among those bearing double-positive alleles was half that of single-positive carriers. These results suggested that, while individuals with a high level of DARC expression (double positive carriers) exhibited low BIAbs responses, those with a lower level of expression (single positive carriers) exhibited a higher inhibitory response. There is no clear explanation for why individuals carrying a single *DARC*-positive allele exhibit a greater level of inhibitory antibodies that those with two positive alleles, especially as there is no evidence that DARC indirectly down-regulates humoral immune responses against the *P. vivax* blood stage, as has been proposed [15]. Because we and others have shown that low DARC erythrocyte expression reduces the risk of *P. vivax* blood-stage infection [34], [35], it can be speculated that limiting access of the *P. vivax* merozoite to the DARC antigen might increase DBPII exposure to the immune system. Considering the quickness of the invasion process [45], [46], and that PvDBP appears to be a poor immunogen that remains sequestered in the micronemes until invasion begins [5], it seems to be a feasible mechanism. Despite this, the levels of erythroid-DARC expression per se might not explain our findings, because, while DARC expression has been found to be lower in persons with FY*B/FY*B than in FY*A/FY*A [47], we found no difference in the inhibitory responses between the FY*A and FY*B homozygotes. Recent evidence suggests that FY*A allelic polymorphism affects the ability of PvDBP antibodies to block parasite invasion [36]. Here, the DBPII/DARC interaction was assessed by using the established COS-7 cyto-adherence assay based upon a multivalent interaction between DBPII on the surface of COS-7 cells and DARC expressed in RBCs [48]. Because, in the COS-7 assay, antibodies are challenged to inhibit a cell interaction that is likely to be highly multivalent [49], it is possible that small differences in antibody activity might be not detected. Studies to assess the BIAbs through an interaction assay of limited valency, such as the recently described flow cytometry based antibody-inhibition assay in which DBPII expressed on the yeast surface interacts with a dimeric recombinant DARC, would be of interest [49]. Although the inhibitory response in the yeast assay seems to be more amenable to high-throughput analysis, the clinical relevance of an assay with low valency interaction remains to be determined. Although these *in vitro* biological assays have made great contributions to the study of the interaction between DBPII and DARC, it is expected that the successful establishment of a long-term culture of *P. vivax* would lead to insights that could improve our understanding of the role of *DARC* genotypes in invasion and immune response processes. Time-lapse microscopy of live *P. vivax* merozoites grown in the presence of inhibitory anti-sera could shed light on this topic.

Through a 12-month follow-up study in the Amazon area, we demonstrated that DBPII inhibitory antibody responses were approximately 50% lower in FY*A/FY*A and FY*B/FY*B double-positive individuals when compared with individuals heterozygous for FY*A or F*B alleles, suggesting a gene-dosage effect. It is not yet clear if this finding has implications for the acquisition of protection against malaria. Due to the relevance of these findings for vaccine development, it would be pertinent to investigate whether such an association exists in other vivax malaria endemic countries.

## Supporting Information

Figure S1 **The DBPII binding to erythrocytes is influenced by *DARC* genotype.** COS-7 cells, transfected with plasmid expressing the gene encoding DBPII, were incubated with erythrocytes from individuals bearing different *DARC* genotypes. The results were expressed as the median of the number of rosettes (5 to 10 samples per genotype) in erythrocyte-binding assays, as described in material and methods. *Box-plots*: solid line across the box is the median, and the bottom and the top of each box represent the 25th and 75th percentiles, respectively; vertical lines represent the range. DARC-negative erythrocytes were used as a negative control. Significant differences were detected between FY*B/FY*B and FY*B/FY*B^ES^ and FY*A/FY*B and FY*B/FY*B^ES^ (P<0.01 by Kruskal–Wallis with Dunn's post hoc test).
(TIF)

Figure S2 **The PvDBP antibody response was not shown to be influenced by acute malaria infection.** The of DBPII and DBPII-IV ELISA-detected antibodies were evaluated at 0, 6, and 12 months, and individuals were grouped according to the presence (yes) or absence (no) of malaria infection at the time of blood collection. The results are shown as frequency (%) of responders (RI>1.0).
(TIF)
